# Cytokine storm modulation in COVID-19: a proposed role for vitamin D and DPP-4 inhibitor combination therapy (VIDPP-4i)

**DOI:** 10.2217/imt-2020-0349

**Published:** 2021-04-28

**Authors:** Marcelo Maia Pinheiro, Andrea Fabbri, Marco Infante

**Affiliations:** ^1^Faculty of Medicine, UNIVAG University Center, Várzea Grande, Mato Grosso, Brazil; ^2^Department of Systems Medicine, Division of Endocrinology & Diabetes, Diabetes Research Institute Federation (DRIF), CTO Hospital, University of Rome Tor Vergata, Rome, Italy; ^3^UniCamillus, Saint Camillus International University of Health Sciences, Section of Endocrinology, Diabetes and Metabolism, Rome, Italy; ^4^Network of Immunity in Infection, Malignancy and Autoimmunity (NIIMA), Universal Scientific Education and Research Network (USERN), Rome, Italy

**Keywords:** cardiovascular disease, COVID-19, cytokine storm, DPP-4 inhibitors, drug repurposing, immunomodulation, SARS-CoV-2, Type 2 diabetes, VIDPP-4i, vitamin D

## Abstract

A dysregulated immune response characterized by the hyperproduction of several pro-inflammatory cytokines (a.k.a. ‘cytokine storm’) plays a central role in the pathophysiology of severe coronavirus disease 2019 (COVID-19) caused by the severe acute respiratory syndrome coronavirus 2 (SARS-CoV-2). In this Perspective article we discuss the evidence for synergistic anti-inflammatory and immunomodulatory properties exerted by vitamin D and dipeptidyl peptidase-4 (DPP-4) inhibitors, the latter being a class of antihyperglycemic agents used for the treatment of Type 2 diabetes, which have also been reported as immunomodulators. Then, we provide the rationale for investigation of vitamin D and DPP-4 inhibitor combination therapy (VIDPP-4i) as an immunomodulation strategy to ratchet down the virulence of SARS-CoV-2, prevent disease progression and modulate the cytokine storm in COVID-19.

## Lay abstract

The so-called ‘cytokine storm’ that drives the hyperproduction of pro-inflammatory mediators, plays a central role in the pathophysiology of severe coronavirus disease 2019 (COVID-19) caused by the severe acute respiratory syndrome coronavirus 2 (SARS-CoV-2). Vitamin D has increasingly been shown to play anti-inflammatory and immunomodulatory properties beyond its role in the regulation of bone homeostasis. Similarly, dipeptidyl peptidase-4 inhibitors (DPP-4i) – a class of antihyperglycemic agents used for the treatment of Type 2 diabetes – have been reported as immunomodulators regardless of their glucose-lowering properties. We, therefore, discuss the role of vitamin D and DPP-4 inhibitor combination therapy (VIDPP-4i) as a potential immunomodulation strategy to prevent the development and/or halt the progression of the COVID-19-induced cytokine storm, particularly in patients with diabetes and cardiovascular disease.

## Tweetable abstract

Vitamin D and DPP-4 inhibitors exert anti-inflammatory and immunomodulatory properties. Vitamin D and DPP-4 inhibitor combination therapy (VIDPP-4i) may represent a valid therapeutic approach to ratchet down the virulence of SARS-CoV-2 and modulate the cytokine storm in COVID-19.

Since the coronavirus disease 2019 (COVID-19) caused by the severe acute respiratory syndrome coronavirus 2 (SARS-CoV-2) was declared a pandemic on March 2020, there has been an unprecedented public health crisis which has placed an enormous strain on healthcare systems worldwide. Older age, cardiovascular disease (CVD), diabetes, obesity, malignancy, chronic lung disease, chronic kidney disease and chronic liver disease have emerged as independent risk factors for adverse clinical outcomes and mortality related to COVID-19 [1–3].

A dysregulated immune response called a ‘cytokine storm’ (also known as ‘cytokine-release syndrome’) and characterized by an excessive increase in circulating levels of several pro-inflammatory cytokines (such as IL-1, IL-2, IL-6, IFN-γ and TNF) has been shown to play a central role in the pathophysiology of the most severe cases of COVID-19, leading to acute respiratory distress syndrome (ARDS), disseminated intravascular coagulation, multiorgan failure and ultimately death [4,5]. A marked activation and exhaustion of CD4^+^ and CD8^+^ T cells accompanied by a skewing of T-cell activation toward T-helper (Th) 17 functional phenotype have been reported in patients with COVID-19 and may contribute to the excessive production of effector pro-inflammatory cytokines such as IL-2, TNF and IFN-γ [4,6]. Patients with severe COVID-19 have higher serum concentration of IL-6, IL-10, IL-2 and IFN-γ, higher numbers of neutrophils in the peripheral blood and reduced counts of T cells (particularly CD8^+^ T cells) compared with patients with mild disease [7]. This suggests that the numbers of neutrophils and T cells, as well as the circulating levels of pro-inflammatory cytokines in the peripheral blood are dynamically correlated with the severity of COVID-19. It is possible that the peripheral lymphopenia – observed especially in patients with severe COVID-19 – reflects the overactivation and functional exhaustion of T cells, as well as the recruitment of lymphocytes to the respiratory tract or adhesion to inflamed respiratory vascular endothelium [8]. Moreover, increased B-cell activation and proliferation have also been correlated with adverse outcomes in severe cases of COVID-19 [9]. Emerging evidence also suggests that endothelial activation and dysfunction contribute to COVID-19 pathogenesis by altering the integrity of vessel barrier, driving a procoagulant state, triggering endothelial inflammation and mediating leukocyte infiltration [10,11].

Diverse SARS-CoV-2 vaccine types are becoming available in different countries and several repurposed drugs have been used for the treatment of COVID-19 since the beginning of the pandemic [12]. Nevertheless, only a few repurposed drugs have been shown to offer a certain degree of effectiveness in preliminary intervention trials [13]. In this Perspective article we propose vitamin D and dipeptidyl peptidase-4 (DPP-4) inhibitors as potential candidates for prevention and modulation of cytokine storm in patients with COVID-19 based on the most recent evidence. DPP-4 inhibitors (DPP-4i) represent a class of oral antihyperglycemic agents used for the treatment of Type 2 diabetes (T2D) [14], which have also been reported to play anti-inflammatory and immunomodulatory properties [15]. References for this manuscript were identified through searches of PubMed using the following terms: ‘vitamin D’, ‘cholecalciferol’, ‘calcifediol’, ‘calcidiol’, ‘dipeptidyl peptidase-4’, ‘DPP-4’ and ‘DPP-4 inhibitors’ in combination with ‘COVID-19’, ‘ACE2’, ‘SARS-CoV-2’, ‘cytokine storm’ and ‘cytokine-release syndrome’. With regard to the literature pertaining to COVID-19, no criteria for publication data were set and we included articles published in English between 1 January 2020 and 28 March 2021. We also checked reference lists in relevant articles and Google Scholar for additional references. Articles resulting from these searches and relevant references cited in those articles were reviewed. The final reference list was generated on the basis of relevance to the topics covered in this publication.

## Vitamin D & COVID-19

Over the last decade, a number of mechanistic studies demonstrated that vitamin D exerts anti-inflammatory and immunomodulatory properties beyond its well-established role in the regulation of bone homeostasis [16,17]. These properties may occur *in vivo* upon the achievement of adequate serum 25-hydroxyvitamin D (25[OH]D) levels, which amount to approximately 40–60 ng/ml [17]. The aforementioned properties are exerted by the biologically active form of vitamin D, which is also referred to as 1,25-dihydroxyvitamin D3 or calcitriol. It has been shown that vitamin D plays a central role in the regulation of innate and adaptive immune responses, promoting antiviral effector mechanisms, reducing the expression of pro-inflammatory cytokines and inducing tolerogenic responses [16–18]. In particular, calcitriol has been shown to: upregulate the transcription of antimicrobial peptides (such as cathelicidin and defensin β2) in various human cell lines (myeloid cells, monocytes/macrophages, neutrophils and keratinocytes); promote the differentiation of monocytes/macrophages and enhance their chemotactic and phagocytic capacity; inhibit the production of several pro-inflammatory cytokines (e.g., IL-6 and TNF-α) by monocytes and macrophages; decrease macrophage antigen-presentation and T-cell stimulatory ability; elicit the shift of macrophage polarization from the M1 pro-inflammatory phenotype (‘classically activated macrophages’) towards the M2 anti-inflammatory phenotype (‘alternatively activated macrophages’); render the dendritic cells more tolerogenic and reduce their antigen-presenting capacity; upregulate regulatory T cells; and favor the shift of T cells from an ‘effector’ pro-inflammatory phenotype toward a ‘regulatory’ anti-inflammatory phenotype by reducing Th1 and Th17 cell differentiation and promoting Th2 cell differentiation [16,17]. Importantly, vitamin D receptor has been identified in almost all immune cells [17], as well as in human airway epithelial cells [19]. The nearly ubiquitous expression of vitamin D receptor enables vitamin D to exert its pleiotropic actions, including the regulation of local ‘respiratory homeostasis’ by upregulating the expression of antimicrobial peptides and/or by directly affecting the replication of respiratory viruses [19]. Vitamin D deficiency (defined as serum 25-hydroxyvitamin D levels less than 20 ng/ml) is a global health issue afflicting more than one billion children and adults worldwide [20]. Since the beginning of COVID-19 pandemic, vitamin D deficiency has been suggested as an independent risk factor for SARS-CoV-2 infection and hospitalization, development of cytokine storm and poor outcomes related to COVID-19 [21–26]. Moreover, the overlap between risk factors for vitamin D deficiency and severe manifestations of COVID-19 (such as Black or Asian ethnic origin, living at higher latitudes, older age and obesity) [27,28] led researchers to consider vitamin D deficiency and COVID-19 as two related pandemics [29]. In a large US retrospective, observational study involving more than 190,000 patients with SARS-CoV-2 results from all 50 states, vitamin D deficiency has been associated with significantly higher SARS-CoV-2 positivity rates [30]. Interestingly, the decrease in SARS-CoV-2 positivity rate associated with 25(OH)D levels appeared to plateau as values approached 55 ng/ml, suggesting that additional benefits exist when 25(OH)D levels are higher than the cut-off value used to define vitamin D sufficiency for bone health (30 ng/ml) [30].

Several observational studies have showed that hospitalized patients with COVID-19 exhibit a markedly high prevalence of hypovitaminosis D, and that vitamin D deficiency is associated with a more advanced disease radiologic stage, along with a significantly higher risk of noninvasive mechanical ventilation and in-hospital mortality [31–35]. It has also been shown that serum vitamin D levels are significantly lower in severe/critical COVID-19 cases compared with mild-to-moderate cases [35]. We recently showed that a lower vitamin D status upon admission is significantly and independently associated with an increased risk of COVID-19-related in-hospital mortality [36]. A systematic review and meta-analysis of observational studies conducted by Pereira *et al.* [37] confirmed a positive association between vitamin D deficiency and COVID-19 severity. In this regard, it is worth mentioning that acute illness and systemic inflammatory response can further lower circulating 25(OH)D levels [38,39], thus explaining, at least in part, the high frequency of severe vitamin D deficiency observed in patients with infectious diseases, including COVID-19 complicated by cytokine release syndrome.

Given the abovementioned anti-inflammatory and immunomodulatory properties of vitamin D, vitamin D deficiency may exacerbate COVID-19 severity and mortality by triggering the hyperinflammatory state and the cytokine storm associated with the most severe cases. Indeed, patients with COVID-19 and vitamin D deficiency have been shown to exhibit significantly higher serum level of several inflammatory and coagulation biomarkers such as C-reactive protein, IL-6, TNF-α, ferritin, fibrinogen and D-dimer [34,36,40]. Therefore, there has been a growing interest in the use of vitamin D as an adjuvant anti-inflammatory and immunomodulatory agent aimed to prevent SARS-CoV-2 infection and/or the progression of COVID-19 toward the severe stage of the disease characterized by the development of cytokine release syndrome [21,41]. Pilot intervention studies investigating the therapeutic role of vitamin D supplementation in COVID-19 yielded promising results. In particular, vitamin D administration (at regular and high doses and in different formulations) has been proven safe and effective in accelerating viral clearance, decreasing fibrinogen levels, reducing the severity of the disease as well as the need for intensive care unit treatment among hospitalized patients (middle-aged and older adults) with COVID-19 [42–45].

Angiotensin-converting enzyme 2 (ACE2), the receptor used by SARS-CoV-2 for cellular entry [46], catalyzes the cleavage of angiotensin II (Ang II) (a vasoconstrictor peptide) into angiotensin 1–7 (Ang-[1–7]) (a vasodilator peptide), thus reducing blood pressure. Moreover, Ang-(1–7) induces nitric oxide synthase (NOS) and further antagonizes Ang II activity via its Ang II type 1 receptor (AT1R) [47]. Following ACE2 receptor binding and SARS-CoV-2 entry into host cells, there is a downregulation of ACE2, resulting in excessive accumulation and pro-inflammatory activity of Ang II potentially accompanied by the development of lung injury, pneumonia, ARDS, myocarditis and/or cardiac injury [48,49]. An analysis on gene expression data from cells in bronchoalveolar lavage fluid from COVID-19 patients has also shown an atypical pattern of the renin-angiotensin system (RAS), which is predicted to elevate bradykinin levels in multiple tissues and promote vasodilation, vascular permeability and hypotension [50]. It has also been suggested that certain polymorphisms in coding, noncoding and regulatory sites of the *ACE2* gene may contribute to the worse clinical course of COVID-19 observed in males and in different regions worldwide [51]. Thus, *ACE2* polymorphisms may partly facilitate or reduce the risk of SARS-CoV-2 infection and adverse outcomes of COVID-19 by affecting the translation regulation and expression of ACE2.

Importantly, vitamin D has been suggested as a negative endocrine modulator of the RAS [47,49]. Of note, vitamin D may decrease the risk of SARS-CoV-2 infection and protect against the symptoms of COVID-19 by inhibiting the synthesis of renin (a proteolytic enzyme and a positive regulator of Ang II) and by increasing ACE2 expression and Ang-(1–7) production in the lung, thus preventing Ang II accumulation, decreasing the pro-inflammatory activity of Ang II and reducing the risk of ARDS, myocarditis and cardiac injury [47,49].

Recently, it has also been suggested that polymorphisms of the vitamin D binding protein (*DBP*) gene may influence circulating 25(OH)D levels and, as a consequence, COVID-19 pathophysiology (in terms of susceptibility to and mortality from SARS-CoV-2 infection). DBP is a serum α-globulin encoded by the *GC* gene and represents the major binding/transport protein of all vitamin D metabolites. Under physiological conditions, the majority of vitamin D metabolites (85–90%) are tightly bound to DBP, whereas only 10–15% of the circulating vitamin D is bound to albumin, and less than 1% represents the unbound (free) form of vitamin D. Besides mediating the transport of vitamin D metabolites and regulating the access of such metabolites to cells and tissues, DBP exerts other important functions such as fatty acid transport, actin scavenging, macrophage activation and chemotaxis [52,53]. Notably, a recent study revealed a significant association between the *DBP1* allele frequency and a lower COVID-19 prevalence and mortality, suggesting that *DBP1* carriers may be less susceptible to SARS-CoV-2 infection and mortality related to COVID-19 [54]. However, further research is needed to better elucidate the relationship between *DBP* and its polymorphisms, vitamin D and COVID-19 pathophysiology.

## Dipeptidyl peptidase-4 (DPP-4), DPP-4 inhibitors & COVID-19

Dipeptidyl peptidase-4 (DPP-4, also referred to as cluster of differentiation 26 or CD26) is a serine exopeptidase existing in two forms: a plasma membrane-bound form (mDPP-4, consisting of a type II transmembrane homodimeric glycoprotein) and a soluble form (sDPP-4). The soluble form maintains its enzymatic (peptidase) activity and is thought to be released from the membrane into the circulation. DPP-4/CD26 is expressed ubiquitously in several cells and tissues, including, but not limited to, kidney, lung, endothelia, liver, intestine and immune cells such as T cells, activated B cells, activated natural killer cells and myeloid cells [55]. DPP-4 has previously been identified as a functional receptor for the spike protein of the Middle East respiratory syndrome coronavirus (MERS-CoV) mediating the virus entry into host cells [56].

DPP-4 inhibitors (DPP-4i, also known as gliptins) have been widely used since 2006 as oral antihyperglycemic agents for the treatment of T2D and have been proven effective in improving glucose control by preventing the DPP-4-mediated cleavage and inactivation of incretin hormones, enhancing endogenous insulin secretion and suppressing glucagon secretion [14]. DPP-4i include sitagliptin, saxagliptin, alogliptin, linagliptin and vildagliptin. In April 2020, we published an article in *CellR4* highlighting the possible therapeutic role of DPP-4 and DPP-4i in COVID-19 pathophysiology [57].

Both SARS-CoV and SARS-CoV-2 use ACE2 as the primary receptor for viral entry into host cells [46]. However, bioinformatics approaches combining human-virus protein interaction prediction, computational model-based selective docking and protein-docking based on crystal structures suggest DPP-4 as a candidate binding target of the receptor-binding S1 domain of the SARS-CoV-2 spike glycoprotein. In addition, the crucial binding residues of DPP-4 are identical to those that are bound to the spike protein of MERS-CoV [58,59]. Since DPP-4 is ubiquitously expressed in several cells and tissues other than lung and respiratory tract, it may therefore participate into the direct SARS-CoV-2-mediated injury of such tissues. Thus, DPP-4 inhibition may have the potential to counteract the DPP-4-mediated SARS-CoV-2 hijacking and virulence and to improve clinical outcomes of COVID-19 by interfering with the interaction between SARS-CoV-2 and target host cells [60–62].

However, the main glucose-independent mechanisms that potentially account for beneficial effects of DPP-4i in COVID-19 include the immunomodulatory, anti-inflammatory and antifibrotic properties exerted by these drugs, which may represent a valid therapeutic tool to prevent or halt the progression toward the hyperinflammatory state and cytokine storm associated with the most severe COVID-19 cases [63–65]. Indeed, a large number of studies demonstrated that DPP-4/CD26 modulates both innate and adaptive immune responses [15,55]. DPP-4/CD26 is expressed only on a fraction of resting T cells, while it becomes significantly upregulated upon T-cell activation [55]. DPP-4/CD26 on T-cell surface induces co-stimulatory effects on T-cell activation, resulting in increased production of Th1 and pro-inflammatory cytokines TNF-α, IFN-γ and IL-6 [66]. CD26-mediated co-stimulation of CD8^+^ T cells exerts a cytotoxic effect primarily via granzyme B, IFN-γ, TNF-α and Fas ligand [67]. In a study conducted by Bengsch *et al.* [68], human Th17 cells producing type 17 cytokines (e.g., IL-17, IL-22, TNF) exhibited the highest levels of enzymatically active DPP-4/CD26 compared with Th1, Th2 and regulatory T cells. Of note, the lowest CD26 expression levels were identified for IL-10-producing CD4^+^ T cells and CD25^hi^CD127^-^FOXP3^+^ regulatory T cells, suggesting suppressive effects exerted by CD26 on such anti-inflammatory cells [68]. Upon activation, approximately 50% of human B cells express DPP-4/CD26 and targeted suppression of DPP-4 activity decreases B-cell activation and DNA synthesis in a dose-dependent manner [55]. Remarkably, a recent large retrospective cohort study conducted on 774,198 patients with T2D showed that the use of DPP-4i is associated with lower risk of autoimmune disorders [69].

DPP-4 inhibition has been shown to attenuate lipopolysaccharide-induced lung injury in murine models of ARDS and to inhibit the release of TNF-α, IL-6 and IL-8 by human lung microvascular endothelial cells [70]. Soare *et al.* [64] recently found that pharmacological inhibition of DPP-4 promoted regression of bleomycin-induced dermal thickness in murine models of systemic sclerosis, and DPP4-knockout mice were less susceptible to bleomycin-induced dermal and pulmonary fibrosis.

Of note, two Italian retrospective observational studies recently showed that the use of DPP-4i at the time of hospital admission in patients with T2D and COVID-19 was associated with reduced mortality, improved clinical outcomes, lower need for noninvasive mechanical ventilation and greater number of hospital discharges [71,72]. In the study conducted by Solerte *et al.* [71], authors included 338 consecutive hospitalized T2D patients with COVID-19 (169 were on standard of care, while 169 were on sitagliptin). Sitagliptin treatment at the time of hospitalization was associated with reduced mortality (hazard ratio: 0.44 [95% CI: 0.29–0.66]; p = 0.0001), along with an improvement in clinical outcomes (60 vs 38% of improved patients; p = 0.0001) and with a higher number of hospital discharges (120 vs 89 of discharged patients; p = 0.0008) compared with patients receiving standard of care, respectively [71]. The study conducted by Mirani *et al.* [72] was a case series involving 387 hospitalized patients with COVID-19; 90 out of 387 participants (23.3%) had T2D. In diabetic patients, the use of DPP-4i was significantly and independently associated with a lower risk of mortality (adjusted hazard ratio: 0.13; 95% CI: 0.02–0.92; p = 0.042) [72]. A recent systematic review and meta-analysis of observational studies found that in-hospital DPP4i use is associated with a significantly reduced COVID-19 mortality in diabetic patients [73]. These findings suggest that diabetic patients with COVID-19 taking DPP-4i may exhibit less severe COVID-19-related pneumonia and end-organ complications.

Additionally, it has been suggested that DPP-4i may exert cardioprotective effects via various mechanisms involving insulin resistance, dysfunctional immunity, oxidative stress, apoptosis, dyslipidemia, adipose tissue dysfunction, as well as antiapoptotic properties of these drugs in the heart and vasculature [74]. A number of studies (mostly preclinical) have shown that DPP-4i can facilitate wound healing, improve endothelial function and prevent the development and progression of atherosclerosis by reducing neutrophil recruitment and activity, attenuating vascular oxidative stress and modulating monocyte/macrophage-mediated responses [15].

Therefore, Du *et al.* [75] have recently proposed DPP-4 inhibition as a potential therapeutic strategy aimed to alleviate the cardiovascular injury (including arrhythmia, acute coronary syndrome and heart failure) caused either directly by SARS-CoV-2 or indirectly by the COVID-19-induced cytokine storm. We previously conducted an *in vitro* study to assess the effects of the DPP-4 inhibitor sitagliptin on human peripheral blood mononuclear cells from healthy volunteers [76]. We demonstrated that sitagliptin is able to markedly reduce the expression of IL-6, IL-17 and IFN-γ and to stimulate the differentiation of Th1 and Th17 cells into TGF-β1-secreting regulatory cells with low CD26 expression [76]. A number of short-term randomized controlled trials demonstrated that sitagliptin exerts anti-inflammatory properties in patients with T2D, resulting in increased expression of IL-10 (an anti-inflammatory cytokine) and reduced expression of various markers of low-grade inflammation, pro-inflammatory cytokines and cell adhesion molecules, such as C-reactive protein, IL-6, IL-18, TNF-α, serum amyloid A-low-density lipoprotein (SAA-LDL) complex, secreted phospholipase-A2 (sPLA2), soluble intercellular adhesion molecule-1 (sICAM-1) and E-selectin [77–79]. A recent Phase II clinical trial published in *The New England Journal of Medicine* showed that sitagliptin in combination with a standard immunosuppressive regimen of tacrolimus and sirolimus resulted in a low incidence of acute graft-versus-host disease by day 100 after myeloablative allogeneic hematopoietic stem-cell transplantation [80].

The anti-inflammatory and immunomodulatory properties of DPP-4i may therefore represent a further advantage in the prevention or management of cytokine storm in COVID-19. An alternative (but not mutually exclusive) explanation for the protective effects of DPP-4i against SARS-CoV-2 infection and progression relies on the hypothesis that pharmacological inhibition of DPP-4 may lead to a significant rise in circulating levels of the soluble form of DPP-4 (sDPP-4) [81], as it has been demonstrated in mice [82]. The subsequent relative abundance of sDPP-4 could offer binding sites for SARS-CoV-2, thus preventing or limiting the attachment of the virus to the membrane-bound DPP-4 (mDPP-4) on target host cells, such as pneumocytes, endothelial cells or other cells relevant for viral spread and replication [81].

## Proposed synergistic effects of vitamin D & DPP-4 inhibitors in COVID-19

Based on the aforementioned remarks, vitamin D and DPP-4i appear to exert synergistic anti-inflammatory and immunomodulatory effects that can be leveraged to ratchet down the virulence of SARS-CoV-2 and prevent the development and/or halt the progression of COVID-19-induced cytokine storm.

Our group [83–87] and other authors [88,89] previously showed that combination therapy with vitamin D and DPP-4i (VIDPP-4i) has the potential ability to protect beta-cell function in autoimmune diabetes. We also reported for the first time a remarkable clinical improvement after 3-year combination therapy with sitagliptin and vitamin D3 in a patient with mononeuritis multiplex, an unusual form of peripheral neuropathy characterized by the presence of perineuritis [90,91]. Recent *in vitro* and *in vivo* studies conducted in patients with T2D demonstrated that VIDPP-4i is able to: reduce the expression of pro-inflammatory cytokines (IFN-γ and IL-17); decrease the proliferation of CD4^+^ T cells and non-CD4^+^ cells; increase the levels of the anti-inflammatory cytokine IL-4; and upregulate the expression of IL-37 and FOXP3 (Forkhead box protein P3), which are well-known markers for regulatory T cells [92–94].

**Figure 1.**
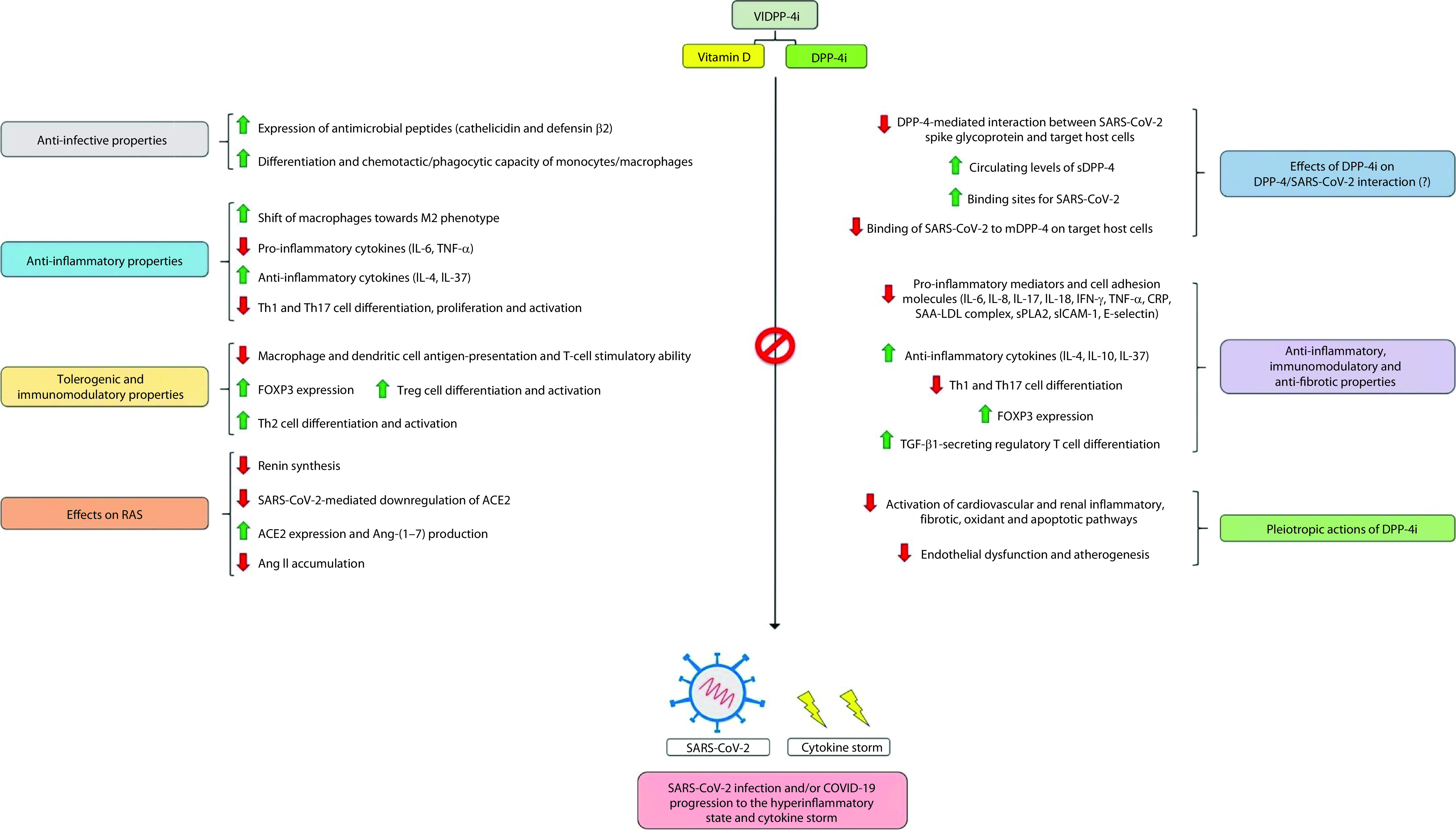
Potential protective effects of VIDPP-4i against SARS-CoV-2 infection and COVID-19 progression to the hyperinflammatory state and cytokine storm. The left side of the figure shows the protective effects of vitamin D, whereas the right side of the figure illustrates the protective effects of DPP-4 inhibitors (DPP-4i). Vitamin D and DPP-4i share many anti-inflammatory and immunomodulatory properties, resulting in synergistic actions of these compounds when they are co-administered. Vitamin D actions refer to those exerted by the biologically active form of vitamin D, which is also known as 1,25-dihydroxyvitamin D3 or calcitriol. ACE2: Angiotensin-converting enzyme 2; Ang-(1–7): Angiotensin 1–7; Ang II: Angiotensin II; COVID-19: Coronavirus disease 2019; CRP: C-reactive protein; DPP-4i: DPP-4 inhibitors; FOXP3: Forkhead box protein P3; IFN-γ: Interferon-gamma; IL: Interleukin; M2: Alternatively activated macrophages; mDPP-4: Membrane-bound DPP-4; RAS: Renin-angiotensin system; SAA-LDL complex: Serum amyloid A-low-density lipoprotein complex; SARS-CoV-2: Severe acute respiratory syndrome coronavirus 2; sDPP-4: Soluble form of DPP-4; sICAM-1: Soluble intercellular adhesion molecule-1; sPLA2: Secreted phospholipase-A2; TGF-β1: Transforming growth factor-beta 1; Th: T helper cell; TNF-α: Tumor necrosis factor-alpha; Treg: Regulatory T cell; VIDPP-4i: Vitamin D and DPP-4 inhibitor combination therapy.

Furthermore, a study conducted in a rat model of fructose/salt-induced insulin resistance showed that the addition of vitamin D3 to the DPP-4i vildagliptin potentiated the renoprotective effects of vildagliptin, which consisted of a series of renal anti-inflammatory, antifibrotic, antioxidant and anti-apoptotic actions [95]. These findings may have additional clinical implications for COVID-19. In fact, chronic kidney disease has emerged as an independent risk factor for poor outcomes related to COVID-19 [1] and SARS-CoV-2 may cause kidney injury through direct cytopathic effects and indirect mechanisms secondary to the cytokine storm and the systemic involvement of the disease [96]. Overall, these findings suggest that vitamin D and DPP-4i administered together exert anti-inflammatory and immunomodulatory actions to a greater extent than vitamin D or DPP-4i administered alone. Moreover, VIDPP-4i may exert protective effects against endothelial dysfunction [97,98], which has been shown to play an important role in COVID-19 pathophysiology [10]. Figure 1 illustrates the potential protective effects of VIDPP-4i against SARS-CoV-2 infection and COVID-19 progression to the hyperinflammatory state and cytokine storm.

## Conclusion

In conclusion, randomized controlled trials are warranted to determine whether VIDPP-4i represents an effective therapeutic intervention for prevention of COVID-19 progression to severe stages and modulation of the cytokine storm, which still represent unmet needs for reducing COVID-19-related hospitalization and mortality rates, particularly in vulnerable and high-risk populations such as diabetic patients.

## Future perspective

Given its proven favorable safety profile (even when administered in high doses) [99], its inexpensive cost and ease of administration, vitamin D supplementation may be a simple therapeutic intervention to prevent disease progression and/or modulate the cytokine storm in COVID-19. On the other hand, DPP-4i exert anti-inflammatory, immunomodulatory and antifibrotic actions beyond their well-known role as glucose-lowering drugs for treatment of T2D. Importantly, DPP-4i are among the noninsulin glucose-lowering agents which have proven to be safe and effective (alone or in combination with insulin therapy) for management of T2D in the hospital [100,101] and outpatient settings [102], even in the context of COVID-19 [103]. This may facilitate the use of DPP-4i in hospitalized patients with COVID-19, without posing additional risks for serious adverse events, including hypoglycemia [102]. Accordingly, practical recommendations for the management of diabetes in patients with COVID-19 do not suggest discontinuation of DPP-4i [104]. A recent systematic review and meta-analysis of the Cardiovascular Outcomes Trials conducted by Grenet *et al.* [105] found that DPP-4i were not associated with either a decreased or an increased risk of overall (non-COVID-19) respiratory tract infection compared with placebo, thus supporting the practical recommendations for the management of diabetes during the COVID-19 pandemic. With this regard, the use of VIDPP-4i may offer a simple and safe means to hamper the hyperinflammatory state and cytokine storm in patients with COVID-19. In addition, the antihyperglycemic properties of DPP-4i, combined with the anti-inflammatory actions of such compounds reported in preclinical and clinical studies of endothelial dysfunction and atherosclerosis, make VIDPP-4i combinatorial approach an intriguing theurapeutic strategy that may be particularly advantageous for patients at high risk for adverse outcomes related to COVID-19, especially for those with comorbid T2D and/or cardiovascular disease and atherosclerosis [106]. Nonetheless, DPP-4i have been investigated in numerous preclinical models of inflammatory diseases, such as arthritis, inflammatory bowel disease and multiple sclerosis [107]. Therefore, the wide repertoire of candidate DPP-4 substrates (e.g., cytokines, chemokines and neuropeptides) and the immunomodulatory actions of DPP-4i suggest that DPP-4i have a broad range of potential therapeutic applications. This may prompt the repurposing of DPP-4i as anti-inflammatory and immunomodulatory agents for the management of different inflammatory diseases, including COVID-19-induced cytokine storm [15,107].

Finally, vitamin D and DPP-4i may also play a role in the prevention of SARS-CoV-2 infection through mechanisms independent of their effects on innate and adaptive immunity. Indeed, vitamin D and DPP-4i might hamper different steps of the viral entry and viral infection cycle of SARS-CoV-2, specifically by interfering with RAS and ACE/Ang II/AT1R axis, and by counteracting the SARS-CoV-2-mediated downregulation of ACE2 expression as well as the interaction between DPP-4 and the receptor-binding S1 domain of the SARS-CoV-2 spike glycoprotein. However, these hypotheses need to be validated by robust mechanistic evidence.

**Executive summary**

**Coronavirus disease 2019-induced cytokine storm**

- A dysregulated immune response called ‘cytokine storm’ occurs in the most severe cases of coronavirus disease 2019 (COVID-19) and consists of an excessive production of pro-inflammatory cytokines.
- The cytokine storm can lead to acute respiratory distress syndrome, disseminated intravascular coagulation, multiorgan failure and ultimately death.
- Even though several repurposed drugs have been used for the treatment of COVID-19, only a few of them have been shown to offer a certain degree of effectiveness in preliminary intervention trials.

**Vitamin D & COVID-19**

- Vitamin D has been shown to exert anti-inflammatory and immunomodulatory properties, promoting antiviral effector mechanisms, reducing the expression of pro-inflammatory cytokines and inducing tolerogenic responses.
- Vitamin D has also been suggested to inhibit the synthesis of renin and to increase the expression of ACE2 and angiotensin 1–7 in the lung, thus preventing angiotensin II (Ang II) accumulation and reducing the Ang II pro-inflammatory activity.
- Observational studies found that a lower vitamin D status correlates with higher severe acute respiratory syndrome coronavirus 2 (SARS-CoV-2) positivity rates in the general population, along with higher levels of pro-inflammatory cytokines and worse prognosis in hospitalized patients with COVID-19.
- Preliminary findings from pilot intervention studies suggest that vitamin D administration may be effective in accelerating the viral clearance and reducing the disease severity in hospitalized patients with COVID-19.

**Dipeptidyl peptidase-4 inhibitors & COVID-19**

- Dipeptidyl peptidase-4 (DPP-4) has been suggested as a candidate binding target of the receptor-binding S1 domain of the SARS-CoV-2 spike glycoprotein.
- DPP-4 inhibitors (DPP-4i, a.k.a. gliptins) are a class of oral antihyperglycemic agents widely used for the treatment of Type 2 diabetes.
- DPP-4 inhibition may have the potential to interfere with the interaction between SARS-CoV-2 and target host cells.
- Emerging evidence suggests that DPP-4 inhibitors exhibit immunomodulatory, anti-inflammatory and antifibrotic properties beyond their well-established role as antihyperglycemic agents.
- Observational studies have recently showed that the use of DPP-4 inhibitors was associated with improved clinical outcomes in hospitalized patients with Type 2 diabetes and COVID-19.

**VIDPP-4i combination therapy & COVID-19**

- Based on emerging evidence coming from studies conducted in autoimmune diabetes, vitamin D and DPP-4 inhibitors appear to exert synergistic anti-inflammatory and immunomodulatory effects.
- According to the aforementioned remarks, vitamin D and DPP-4 inhibitor combination therapy (VIDPP-4i) may represent a valid therapeutic approach to ratchet down the virulence of SARS-CoV-2, prevent disease progression and modulate the cytokine storm in COVID-19, particularly in patients at high risk for COVID-19-related adverse outcomes such as those with comorbid diabetes, cardiovascular disease and/or atherosclerosis.
- VIDPP-4i may also play a role in the prevention of SARS-CoV-2 infection by interfering with renin-angiotensin system and ACE/Ang II/AT1R axis, and counteracting the SARS-CoV-2-mediated downregulation of ACE2 as well as the interaction between DPP-4 and the SARS-CoV-2 spike glycoprotein.
- Randomized controlled trials are needed to establish whether VIDPP-4i represents a safe and effective intervention for prevention of SARS-CoV-2 infection and COVID-19 progression to the cytokine storm.

## References

[1] Noor FM, Islam MM. Prevalence and associated risk factors of mortality among COVID-19 patients: a meta-analysis. J. Community Health 45(6), 1270–1282 (2020). 32918645 10.1007/s10900-020-00920-x PMC7486583

[2] Roy S, Mazumder T, Banik S. The association of cardiovascular diseases and diabetes mellitus with COVID-19 (SARS-CoV-2) and their possible mechanisms. SN. Compr. Clin. Med. 1–6 (2020) (Epub ahead of print). 10.1007/s42399-020-00376-z PMC7316523 32838148

[3] Drucker DJ. Coronavirus infections and Type 2 diabetes-shared pathways with therapeutic implications. Endocr. Rev. 41(3), bnaa011 (2020). 32294179 10.1210/endrev/bnaa011 PMC7184382

[4] Mangalmurti N, Hunter CA. Cytokine storms: understanding COVID-19. Immunity 53(1), 19–25 (2020). 32610079 10.1016/j.immuni.2020.06.017 PMC7321048

[5] Ragab D, Salah Eldin H, Taeimah M, Khattab R, Salem R. The COVID-19 cytokine storm; what we know so far. Front. Immunol. 11, 1446 (2020). 32612617 10.3389/fimmu.2020.01446 PMC7308649

[6] De Biasi S, Meschiari M, Gibellini L Marked T cell activation, senescence, exhaustion and skewing towards TH17 in patients with COVID-19 pneumonia. Nat. Commun. 11(1), 3434 (2020). 32632085 10.1038/s41467-020-17292-4 PMC7338513

[7] Liu J, Li S, Liang B Longitudinal characteristics of lymphocyte responses and cytokine profiles in the peripheral blood of SARS-CoV-2 infected patients. EBioMedicine 55, 102763 (2020). 32361250 10.1016/j.ebiom.2020.102763 PMC7165294

[8] Chen Z, John Wherry E. T cell responses in patients with COVID-19. Nat. Rev. Immunol. 20(9), 529–536 (2020). 32728222 10.1038/s41577-020-0402-6 PMC7389156

[9] Yang L, Liu S, Liu J COVID-19: immunopathogenesis and immunotherapeutics. Signal Transduct. Target. Ther. 5(1), 128 (2020). 32712629 10.1038/s41392-020-00243-2 PMC7381863

[10] Jin Y, Ji W, Yang H, Chen S, Zhang W, Duan G. Endothelial activation and dysfunction in COVID-19: from basic mechanisms to potential therapeutic approaches. Signal Transduct. Target. Ther. 5(1), 293 (2020). 33361764 10.1038/s41392-020-00454-7 PMC7758411

[11] Sinha P, Matthay MA, Calfee CS. Is a “cytokine storm” relevant to COVID-19? JAMA Intern. Med. 180(9), 1152–1154 (2020). 32602883 10.1001/jamainternmed.2020.3313

[12] Sultana J, Crisafulli S, Gabbay F, Lynn E, Shakir S, Trifirò G. Challenges for drug repurposing in the COVID-19 pandemic era. Front. Pharmacol. 11, 588654 (2020). 33240091 10.3389/fphar.2020.588654 PMC7677570

[13] Lenze EJ, Mattar C, Zorumski CF Fluvoxamine vs placebo and clinical deterioration in outpatients with symptomatic COVID-19: a randomized clinical trial. JAMA 324(22), 2292–2300 (2020). 33180097 10.1001/jama.2020.22760 PMC7662481

[14] Dicker D. DPP-4 inhibitors: impact on glycemic control and cardiovascular risk factors. Diabetes Care 34(Suppl. 2), S276–S278 (2011). 21525468 10.2337/dc11-s229 PMC3632192

[15] Yazbeck R, Jaenisch SE, Abbott CA. Dipeptidyl peptidase 4 inhibitors: applications in innate immunity? Biochem. Pharmacol. 188, 114517 (2021) (Epub ahead of print). 33722535 10.1016/j.bcp.2021.114517 PMC7954778

[16] Caprio M, Infante M, Calanchini M, Mammi C, Fabbri A. Vitamin D: not just the bone. Evidence for beneficial pleiotropic extraskeletal effects. Eat. Weight Disord. 22(1), 27–41 (2017). 27553017 10.1007/s40519-016-0312-6

[17] Fabbri A, Infante M, Ricordi C. Editorial - vitamin D status: a key modulator of innate immunity and natural defense from acute viral respiratory infections. Eur. Rev. Med. Pharmacol. Sci. 24(7), 4048–4052 (2020). 32329882 10.26355/eurrev_202004_20876

[18] Infante M, Ricordi C, Padilla N The role of vitamin D and omega-3 PUFAs in islet transplantation. Nutrients 11(12), 2937 (2019). 10.3390/nu11122937 PMC6950335 31816979

[19] Zdrenghea MT, Makrinioti H, Bagacean C, Bush A, Johnston SL, Stanciu LA. Vitamin D modulation of innate immune responses to respiratory viral infections. Rev. Med. Virol. 27(1), (2017). 10.1002/rmv.1909 27714929

[20] Holick MF. The vitamin D deficiency pandemic: approaches for diagnosis, treatment and prevention. Rev. Endocr. Metab. Disord. 18(2), 153–165 (2017). 28516265 10.1007/s11154-017-9424-1

[21] Grant WB, Lahore H, McDonnell SL Evidence that vitamin D supplementation could reduce risk of influenza and COVID-19 infections and deaths. Nutrients 12(4), 988 (2020). 10.3390/nu12061620 PMC7352449 32492787

[22] Glinsky GV. Tripartite combination of candidate pandemic mitigation agents: vitamin D, quercetin, and estradiol manifest properties of medicinal agents for targeted mitigation of the COVID-19 pandemic defined by genomics-guided tracing of SARS-CoV-2 targets in human cells. Biomedicines 8(5), 129 (2020). 10.3390/biomedicines8050129 PMC7277789 32455629

[23] Bilezikian JP, Bikle D, Hewison M Mechanisms in endocrinology: vitamin D and COVID-19. Eur. J. Endocrinol. 183(5), R133–R147 (2020). 32755992 10.1530/EJE-20-0665 PMC9494342

[24] Ali N. Role of vitamin D in preventing of COVID-19 infection, progression and severity. J. Infect. Public Health 13(10), 1373–1380 (2020). 32605780 10.1016/j.jiph.2020.06.021 PMC7305922

[25] Meltzer DO, Best TJ, Zhang H, Vokes T, Arora V, Solway J. Association of vitamin D status and other clinical characteristics with COVID-19 test results. JAMA Netw. Open 3(9), e2019722 (2020). 32880651 10.1001/jamanetworkopen.2020.19722 PMC7489852

[26] Merzon E, Tworowski D, Gorohovski A Low plasma 25(OH) vitamin D level is associated with increased risk of COVID-19 infection: an Israeli population-based study. FEBS J. 287(17), 3693–3702 (2020). 32700398 10.1111/febs.15495 PMC7404739

[27] Whittemore PB. COVID-19 fatalities, latitude, sunlight, and vitamin D. Am. J. Infect. Control 48(9), 1042–1044 (2020). 32599103 10.1016/j.ajic.2020.06.193 PMC7319635

[28] Martineau AR, Forouhi NG. Vitamin D for COVID-19: a case to answer? Lancet Diabetes Endocrinol. 8(9), 735–736 (2020). 32758429 10.1016/S2213-8587(20)30268-0 PMC7398646

[29] Kara M, Ekiz T, Ricci V, Kara Ö, Chang KV, Özçakar L. ‘Scientific strabismus’ or two related pandemics: coronavirus disease and vitamin D deficiency. Br. J. Nutr. 124(7), 736–741 (2020). 32393401 10.1017/S0007114520001749 PMC7300194

[30] Kaufman HW, Niles JK, Kroll MH, Bi C, Holick MF. SARS-CoV-2 positivity rates associated with circulating 25-hydroxyvitamin D levels. PLoS ONE 15(9), e0239252 (2020). 32941512 10.1371/journal.pone.0239252 PMC7498100

[31] Carpagnano GE, Di Lecce V, Quaranta VN Vitamin D deficiency as a predictor of poor prognosis in patients with acute respiratory failure due to COVID-19. J. Endocrinol. Invest. 44(4), 765–771 (2020) (Epub ahead of print). 32772324 10.1007/s40618-020-01370-x PMC7415009

[32] Radujkovic A, Hippchen T, Tiwari-Heckler S, Dreher S, Boxberger M, Merle U. Vitamin D deficiency and outcome of COVID-19 patients. Nutrients 12(9), 2757 (2020). 10.3390/nu12092757 PMC7551780 32927735

[33] De Smet D, De Smet K, Herroelen P, Gryspeerdt S, Martens GA. Serum 25(OH)D level on hospital admission associated with COVID-19 stage and mortality. Am. J. Clin. Pathol. 155(3), 381–388 (2020). 10.1093/ajcp/aqaa252 PMC7717135 33236114

[34] Hernández JL, Nan D, Fernandez-Ayala M Vitamin D status in hospitalized patients with SARS-CoV-2 Infection. J. Clin. Endocrinol. Metab.(2020) (Epub ahead of print). https://doi.org/doi:10.1210/clinem/dgaa733 10.1210/clinem/dgaa733 PMC7797757 33159440

[35] Ye K, Tang F, Liao X Does serum vitamin D level affect COVID-19 infection and its severity?-A case-control study. J. Am. Coll. Nutr. 1–8 (2020) (Epub ahead of print). 10.1080/07315724.2020.1826005 33048028

[36] Infante M, Buoso A, Pieri M Low vitamin D status at admission as a risk factor for poor survival in hospitalized patients with COVID-19: an Italian retrospective study. J. Am. Coll. Nutr. 1–16 (2021) (Epub ahead of print). 10.1080/07315724.2021.1877580 PMC7899172 33600292

[37] Pereira M, Dantas Damascena A, Galvão Azevedo LM, de Almeida Oliveira T, da Mota Santana J. Vitamin D deficiency aggravates COVID-19: systematic review and meta-analysis. Crit. Rev. Food Sci. Nutr. 1–9 (2020) (Epub ahead of print). 10.1080/10408398.2020.1841090 33146028

[38] French CB, McDonnell SL, Vieth R. 25-Hydroxyvitamin D variability within-person due to diurnal rhythm and illness: a case report. J. Med. Case Rep. 13(1), 29 (2019). 30712514 10.1186/s13256-018-1948-9 PMC6360762

[39] Smolders J, van den Ouweland J, Geven C, Pickkers P, Kox M. Letter to the Editor: vitamin D deficiency in COVID-19: mixing up cause and consequence. Metabolism 115, 154434 (2021). 33217408 10.1016/j.metabol.2020.154434 PMC7671645

[40] Jain A, Chaurasia R, Sengar NS, Singh M, Mahor S, Narain S. Analysis of vitamin D level among asymptomatic and critically ill COVID-19 patients and its correlation with inflammatory markers. Sci. Rep. 10(1), 20191 (2020). 33214648 10.1038/s41598-020-77093-z PMC7677378

[41] Jakovac H. COVID-19 and vitamin D-is there a link and an opportunity for intervention? Am. J. Physiol. Endocrinol. Metab. 318(5), E589 (2020). 32297519 10.1152/ajpendo.00138.2020 PMC7191631

[42] Entrenas Castillo M, Entrenas Costa LM, Vaquero Barrios JM “Effect of calcifediol treatment and best available therapy versus best available therapy on intensive care unit admission and mortality among patients hospitalized for COVID-19: a pilot randomized clinical study”. J. Steroid Biochem. Mol. Biol. 203, 105751 (2020). 32871238 10.1016/j.jsbmb.2020.105751 PMC7456194

[43] Rastogi A, Bhansali A, Khare N Short term, high-dose vitamin D supplementation for COVID-19 disease: a randomised, placebo-controlled, study (SHADE study). Postgrad. Med. J. (2020) (Epub ahead of print). http://dx.doi.org/doi:10.1136/postgradmedj-2020-139065 10.1136/postgradmedj-2020-139065 33184146

[44] Annweiler G, Corvaisier M, Gautier J Vitamin D supplementation associated to better survival in hospitalized frail elderly COVID-19 patients: the GERIA-COVID quasi-experimental study. Nutrients 12(11), 3377 (2020). 10.3390/nu12113377 PMC7693938 33147894

[45] Annweiler C, Hanotte B, Grandin de l'Eprevier C, Sabatier JM, Lafaie L, Célarier T. Vitamin D and survival in COVID-19 patients: a quasi-experimental study. J. Steroid Biochem. Mol. Biol. 204, 105771 (2020). 33065275 10.1016/j.jsbmb.2020.105771 PMC7553119

[46] Bourgonje AR, Abdulle AE, Timens W Angiotensin-converting enzyme 2 (ACE2), SARS-CoV-2 and the pathophysiology of coronavirus disease 2019 (COVID-19). J. Pathol. 251(3), 228–248 (2020). 32418199 10.1002/path.5471 PMC7276767

[47] Malek Mahdavi A. A brief review of interplay between vitamin D and angiotensin-converting enzyme 2: implications for a potential treatment for COVID-19. Rev. Med. Virol. 30(5), e2119 (2020). 32584474 10.1002/rmv.2119 PMC7362103

[48] Hanff TC, Harhay MO, Brown TS, Cohen JB, Mohareb AM. Is there an association between COVID-19 mortality and the renin-angiotensin system? A call for epidemiologic investigations. Clin. Infect. Dis. 71(15), 870–874 (2020). 32215613 10.1093/cid/ciaa329 PMC7184340

[49] Aygun H. Vitamin D can prevent COVID-19 infection-induced multiple organ damage. Naunyn Schmiedebergs Arch. Pharmacol. 393(7), 1157–1160 (2020). 32451597 10.1007/s00210-020-01911-4 PMC7246956

[50] Garvin MR, Alvarez C, Miller JI A mechanistic model and therapeutic interventions for COVID-19 involving a RAS-mediated bradykinin storm. Elife 9, e59177 (2020). 32633718 10.7554/eLife.59177 PMC7410499

[51] Khayat AS, de Assumpção PP, Meireles Khayat BC ACE2 polymorphisms as potential players in COVID-19 outcome. PLoS ONE 15(12), e0243887 (2020). 33370311 10.1371/journal.pone.0243887 PMC7769452

[52] Speeckaert M, Huang G, Delanghe JR, Taes YE. Biological and clinical aspects of the vitamin D binding protein (Gc-globulin) and its polymorphism. Clin. Chim. Acta 372(1–2), 33–42 (2006). 16697362 10.1016/j.cca.2006.03.011

[53] Speeckaert MM, Speeckeart R, Delanghe JR. Genetic polymorphisms, vitamin D binding protein and vitamin D deficiency in COVID-19. Eur. Respir. J. 2004638 (2021) (Epub ahead of print). 33542051 10.1183/13993003.04638-2020 PMC7861048

[54] Speeckaert MM, De Buyzere ML, Delanghe JR. Vitamin D binding protein polymorphism and COVID-19. J. Med. Virol. 93(2), 705–707 (2021). 32918506 10.1002/jmv.26508

[55] Klemann C, Wagner L, Stephan M, von Hörsten S. Cut to the chase: a review of CD26/dipeptidyl peptidase-4's (DPP4) entanglement in the immune system. Clin. Exp. Immunol. 185(1), 1–21 (2016). 26919392 10.1111/cei.12781 PMC4908298

[56] Raj VS, Mou H, Smits SL Dipeptidyl peptidase 4 is a functional receptor for the emerging human coronavirus-EMC. Nature 495(7440), 251–254 (2013). 23486063 10.1038/nature12005 PMC7095326

[57] Maia Pinheiro M, Monteiro Pinheiro J, Moura Maia Pinheiro F. Editorial – COVID-19 pandemic: is it time to learn about DPP-4/CD26? CellR4 8, e2835 (2020).

[58] Li Y, Zhang Z, Yang L The MERS-CoV receptor DPP4 as a candidate binding target of the SARS-CoV-2 spike. iScience 23(6), 101160 (2020). 32405622 10.1016/j.isci.2020.101160 PMC7219414

[59] Vankadari N, Wilce JA. Emerging WuHan (COVID-19) coronavirus: glycan shield and structure prediction of spike glycoprotein and its interaction with human CD26. Emerg. Microbes Infect. 9(1), 601–604 (2020). 32178593 10.1080/22221751.2020.1739565 PMC7103712

[60] Solerte SB, Di Sabatino A, Galli M, Fiorina P. Dipeptidyl peptidase-4 (DPP4) inhibition in COVID-19. Acta Diabetol. 57(7), 779–783 (2020). 32506195 10.1007/s00592-020-01539-z PMC7275134

[61] Chen CF, Chien CH, Yang YP Role of dipeptidyl peptidase-4 inhibitors in patients with diabetes infected with coronavirus-19. J. Chin. Med. Assoc. 83(8), 710–711 (2020). 32349031 10.1097/JCMA.0000000000000338 PMC7493766

[62] Bassendine MF, Bridge SH, McCaughan GW, Gorrell MD. COVID-19 and comorbidities: a role for dipeptidyl peptidase 4 (DPP4) in disease severity? J. Diabetes 12(9), 649–658 (2020). 32394639 10.1111/1753-0407.13052

[63] Strollo R, Pozzilli P. DPP4 inhibition: preventing SARS-CoV-2 infection and/or progression of COVID-19? Diabetes Metab. Res. Rev. 36(8), e3330 (2020). 32336007 10.1002/dmrr.3330 PMC7267128

[64] Soare A, Györfi HA, Matei AE Dipeptidylpeptidase 4 as a marker of activated fibroblasts and a potential target for the treatment of fibrosis in systemic sclerosis. Arthritis Rheumatol. 72(1), 137–149 (2020). 31350829 10.1002/art.41058

[65] Iacobellis G. COVID-19 and diabetes: can DPP4 inhibition play a role? Diabetes Res. Clin. Pract. 162, 108125 (2020). 32224164 10.1016/j.diabres.2020.108125 PMC7271223

[66] Pacheco R, Martinez-Navio JM, Lejeune M CD26, adenosine deaminase, and adenosine receptors mediate costimulatory signals in the immunological synapse. Proc. Natl Acad. Sci. USA 102(27), 9583–9588 (2005). 15983379 10.1073/pnas.0501050102 PMC1172240

[67] Hatano R, Ohnuma K, Yamamoto J, Dang NH, Morimoto C. CD26-mediated co-stimulation in human CD8(+) T cells provokes effector function via pro-inflammatory cytokine production. Immunology 138(2), 165–172 (2013). 23113658 10.1111/imm.12028 PMC3575769

[68] Bengsch B, Seigel B, Flecken T, Wolanski J, Blum HE, Thimme R. Human Th17 cells express high levels of enzymatically active dipeptidylpeptidase IV (CD26). J. Immunol. 188(11), 5438–5447 (2012). 22539793 10.4049/jimmunol.1103801

[69] Chen YC, Chen TH, Sun CC Dipeptidyl peptidase-4 inhibitors and the risks of autoimmune diseases in Type 2 diabetes mellitus patients in Taiwan: a nationwide population-based cohort study. Acta Diabetol. 57(10), 1181–1192 (2020). 32318876 10.1007/s00592-020-01533-5 PMC7173685

[70] Kawasaki T, Chen W, Htwe YM, Tatsumi K, Dudek SM. DPP4 inhibition by sitagliptin attenuates LPS-induced lung injury in mice. Am. J. Physiol. Lung Cell. Mol. Physiol. 315(5), L834–L845 (2018). 30188745 10.1152/ajplung.00031.2018

[71] Solerte SB, D'Addio F, Trevisan R Sitagliptin treatment at the time of hospitalization was associated with reduced mortality in patients with Type 2 diabetes and COVID-19: a multicenter, case-control, retrospective, observational study. Diabetes Care 43(12), 2999–3006 (2020). 32994187 10.2337/dc20-1521 PMC7770266

[72] Mirani M, Favacchio G, Carrone F Impact of comorbidities and glycemia at admission and dipeptidyl peptidase 4 inhibitors in patients with Type 2 diabetes with COVID-19: a case series from an academic hospital in Lombardy, Italy. Diabetes Care 43(12), 3042–3049 (2020). 33023989 10.2337/dc20-1340

[73] Pal R, Banerjee M, Mukherjee S, Singh Bhogal R, Kaur A, Bhadada SK. Dipeptidyl peptidase-4 inhibitor use and mortality in COVID-19 patients with diabetes mellitus: an updated systematic review and meta-analysis. Ther. Adv. Endocrinol. Metab. 12, 2042018821996482 (2021) (Epub ahead of print). 33680425 10.1177/2042018821996482 PMC7897812

[74] Aroor AR, Sowers JR, Jia G, DeMarco VG. Pleiotropic effects of the dipeptidylpeptidase-4 inhibitors on the cardiovascular system. Am. J. Physiol. Heart Circ. Physiol. 307(4), H477–H492 (2014). 24929856 10.1152/ajpheart.00209.2014 PMC4137125

[75] Du H, Wang DW, Chen C. The potential effects of DPP-4 inhibitors on cardiovascular system in COVID-19 patients. J. Cell. Mol. Med. 24(18), 10274–10278 (2020). 32713161 10.1111/jcmm.15674 PMC7521316

[76] Pinheiro MM, Stoppa CL, Valduga CJ Sitagliptin inhibit human lymphocytes proliferation and Th1/Th17 differentiation *in vitro*. Eur. J. Pharm. Sci. 100, 17–24 (2017). 28065853 10.1016/j.ejps.2016.12.040

[77] Makdissi A, Ghanim H, Vora M Sitagliptin exerts an antinflammatory action. J. Clin. Endocrinol. Metab. 97(9), 3333–3341 (2012). 22745245 10.1210/jc.2012-1544 PMC3431580

[78] Satoh-Asahara N, Sasaki Y, Wada H A dipeptidyl peptidase-4 inhibitor, sitagliptin, exerts anti-inflammatory effects in Type 2 diabetic patients. Metabolism 62(3), 347–351 (2013). 23062489 10.1016/j.metabol.2012.09.004

[79] Tremblay AJ, Lamarche B, Deacon CF, Weisnagel SJ, Couture P. Effects of sitagliptin therapy on markers of low-grade inflammation and cell adhesion molecules in patients with type 2 diabetes. Metabolism 63(9), 1141–1148 (2014). 25034387 10.1016/j.metabol.2014.06.004

[80] Farag SS, Abu Zaid M, Schwartz JE Dipeptidyl peptidase 4 inhibition for prophylaxis of acute graft-versus-host disease. N. Engl. J. Med. 384(1), 11–19 (2021). 33406328 10.1056/NEJMoa2027372 PMC7845486

[81] Nauck MA, Meier JJ. Reduced COVID-19 mortality with sitagliptin treatment? Weighing the dissemination of potentially lifesaving findings against the assurance of high scientific standards. Diabetes Care 43(12), 2906–2909 (2020). 33033068 10.2337/dci20-0062

[82] Varin EM, Mulvihill EE, Beaudry JL Circulating levels of soluble dipeptidyl peptidase-4 are dissociated from inflammation and induced by enzymatic DPP4 inhibition. Cell. Metab. 29(2), 320–334.e5 (2019). 30393019 10.1016/j.cmet.2018.10.001

[83] Pinheiro MM, Pinheiro FM, Torres MA. Four-year clinical remission of Type 1 diabetes mellitus in two patients treated with sitagliptin and vitamin D3. Endocrinol. Diabetes Metab. Case Rep. 2016, 16–0099 (2016). 10.1530/EDM-16-0099 PMC5184778 28035286

[84] Pinheiro MM, Pinheiro FMM, Trabachin ML. Dipeptidyl peptidase-4 inhibitors (DPP-4i) combined with vitamin D3: an exploration to treat new-onset Type 1 diabetes mellitus and latent autoimmune diabetes in adults in the future. Int. Immunopharmacol. 57, 11–17 (2018). 29452961 10.1016/j.intimp.2018.02.003

[85] Pinheiro M, Pinheiro F. Prolonging the honeymoon phase in T1DM with sitagliptin plus vitamin D3. The Official Journal of ATTD Advanced Technologies & Treatments for Diabetes Conference Madrid, Spain. Diabetes Technol. Ther. 22(S1), A1–A250 (2020). 32069144 10.1089/dia.2020.2525.abstracts

[86] Infante M, Ricordi C, Sanchez J Influence of vitamin D on islet autoimmunity and beta-cell function in Type 1 diabetes. Nutrients 11(9), 2185 (2019). 10.3390/nu11092185 PMC6769474 31514368

[87] Pinheiro MM, Pinheiro FMM, Diniz SN, Fabbri A, Infante M. Combination of vitamin D and dipeptidyl peptidase-4 inhibitors (VIDPP-4i) as an immunomodulation therapy for autoimmune diabetes. Int. Immunopharmacol. 95, 107518 (2021). 33756226 10.1016/j.intimp.2021.107518

[88] Rapti E, Karras S, Grammatiki M Combined treatment with sitagliptin and vitamin D in a patient with latent autoimmune diabetes in adults. Endocrinol. Diabetes Metab. Case Rep. 2016, 150136 (2016). 27252860 10.1530/EDM-15-0136 PMC4888610

[89] Zhang Z, Yan X, Wu C Adding vitamin D3 to the dipeptidyl peptidase-4 inhibitor saxagliptin has the potential to protect β-cell function in LADA patients: a 1-year pilot study. Diabetes Metab. Res. Rev. 36(5), e3298 (2020). 32043288 10.1002/dmrr.3298

[90] Maia Pinheiro M, Moura Maia Pinheiro F, Pires Amaral Resende LL, Nogueira Diniz S, Fabbri A, Infante M. Improvement of pure sensory mononeuritis multiplex and IgG1 deficiency with sitagliptin plus Vitamin D3. Eur. Rev. Med. Pharmacol. Sci. 24(15), 8151–8159 (2020). 32767343 10.26355/eurrev_202008_22502

[91] Maia Pinheiro M, Maia Pinheiro FM, Amaral Resende LLP, Diniz SN, Fabbri A, Infante M. 36-month follow-up of a pure sensory mononeuritis multiplex and IgG1 deficiency improved after treatment with sitagliptin and Vitamin D3. Eur. Rev. Med. Pharmacol. Sci. 25(4), 1768–1769 (2021). 33660837 10.26355/eurrev_202102_25064

[92] Mahabadi-Ashtiyani E, Sheikh V, Borzouei S, Salehi I, Alahgholi-Hajibehzad M. The increased T helper cells proliferation and inflammatory responses in patients with Type 2 diabetes mellitus is suppressed by sitagliptin and vitamin D3 *in vitro*. Inflamm. Res. 68(10), 857–866 (2019). 31236602 10.1007/s00011-019-01265-5

[93] Telikani Z, Sheikh V, Zamani A Effects of sitagliptin and vitamin D3 on T helper cell transcription factors and cytokine production in clinical subgroups of Type 2 diabetes mellitus: highlights upregulation of FOXP3 and IL-37. Immunopharmacol. Immunotoxicol. 41(2), 299–311 (2019). 30907193 10.1080/08923973.2019.1593447

[94] Borzouei S, Sheikh V, Ghasemi M Anti-Inflammatory effect of combined sitagliptin and vitamin D3 on cytokines profile in patients with Type 2 diabetes mellitus. J. Interferon Cytokine Res. 39(5), 293–301 (2019). 30855208 10.1089/jir.2018.0144

[95] Wahba NS, Abdel-Ghany RH, Ghareib SA, Abdel-Aal M, Alsemeh AE. Vitamin D3 potentiates the renoprotective effects of vildagliptin in a rat model of fructose/salt-induced insulin resistance. Eur. J. Pharm. Sci. 144, 105196 (2020). 31866564 10.1016/j.ejps.2019.105196

[96] Fortarezza F, Pezzuto F. COVID-19 nephropathy: what could pathologist say? J. Nephropathol. 9(4), e32 (2020).

[97] Caprio M, Mammi C, Rosano GM. Vitamin D: a novel player in endothelial function and dysfunction. Arch. Med. Sci. 8(1), 4–5 (2012). 22457665 10.5114/aoms.2012.27271 PMC3309427

[98] Aini K, Fukuda D, Tanaka K Vildagliptin, a DPP-4 Inhibitor, attenuates endothelial dysfunction and atherogenesis in nondiabetic apolipoprotein E-deficient mice. Int. Heart J. 60(6), 1421–1429 (2019). 31735774 10.1536/ihj.19-117

[99] Fassio A, Adami G, Rossini M Pharmacokinetics of oral cholecalciferol in healthy subjects with vitamin D deficiency: a randomized open-label study. Nutrients 12(6), 1553 (2020). 10.3390/nu12061553 PMC7352201 32471106

[100] American Diabetes Association. 15. Diabetes care in the hospital: standards of medical care in diabetes – 2021. Diabetes Care 44(Suppl. 1), S211–S220 (2021). 33298426 10.2337/dc21-S015

[101] Lorenzo-González C, Atienza-Sánchez E, Reyes-Umpierrez D Safety and efficacy of DPP4-inhibitors for management of hospitalized general medicine and surgery patients with Type 2 diabetes. Endocr. Pract. 26(7), 722–728 (2020) (Epub ahead of print). 33471640 10.4158/EP-2019-0481 PMC11305855

[102] Ling J, Cheng P, Ge L The efficacy and safety of dipeptidyl peptidase-4 inhibitors for Type 2 diabetes: a Bayesian network meta-analysis of 58 randomized controlled trials. Acta Diabetol. 56(3), 249–272 (2019). 30242726 10.1007/s00592-018-1222-z

[103] Roussel R, Darmon P, Pichelin M Use of dipeptidyl peptidase-4 inhibitors and prognosis of COVID-19 in hospitalized patients with Type 2 diabetes: a propensity score analysis from the CORONADO study. Diabetes Obes. Metab. (2021) (Epub ahead of print). 10.1111/dom.14324 PMC8013481 33528920

[104] Bornstein SR, Rubino F, Khunti K Practical recommendations for the management of diabetes in patients with COVID-19. Lancet Diabetes Endocrinol. 8(6), 546–550 (2020). 32334646 10.1016/S2213-8587(20)30152-2 PMC7180013

[105] Grenet G, Mekhaldi S, Mainbourg S DPP-4 inhibitors and respiratory infection: a systematic review and meta-analysis of the cardiovascular outcomes trials. Diabetes Care 44(3), e36–e37 (2021). 33436399 10.2337/dc20-2018

[106] de Almeida-Pititto B, Dualib PM, Zajdenverg L Severity and mortality of COVID 19 in patients with diabetes, hypertension and cardiovascular disease: a meta-analysis. Diabetol. Metab. Syndr. 12, 75 (2020). 32874207 10.1186/s13098-020-00586-4 PMC7456786

[107] Yazbeck R, Howarth GS, Abbott CA. Dipeptidyl peptidase inhibitors, an emerging drug class for inflammatory disease? Trends Pharmacol. Sci. 30(11), 600–607 (2009). 19837468 10.1016/j.tips.2009.08.003

